# Health Care Burden and Cost Associated with Fetal Alcohol Syndrome: Based on Official Canadian Data

**DOI:** 10.1371/journal.pone.0043024

**Published:** 2012-08-10

**Authors:** Svetlana Popova, Shannon Lange, Larry Burd, Jürgen Rehm

**Affiliations:** 1 Social and Epidemiological Research Department, Centre for Addiction and Mental Health, Toronto, Ontario, Canada; 2 Dalla Lana School of Public Health, University of Toronto, Toronto, Ontario, Canada; 3 Factor-Inwentash Faculty of Social Work, University of Toronto, West Toronto, Ontario, Canada; 4 Department of Pediatrics, University of North Dakota School of Medicine and Health Sciences, Grand Forks, North Dakota, United States of America; 5 Epidemiological Research Unit, Klinische Psychologie and Psychotherapie, Technische Universität Dresden, Dresden, Germany; Partners for Applied Social Sciences (PASS), Belgium

## Abstract

**Background:**

Fetal Alcohol Spectrum Disorder (FASD) is a group of disorders caused by prenatal alcohol exposure. From this group, Fetal Alcohol Syndrome (FAS) is the only disorder coded in the *International Classification of Diseases*, version 10 (ICD-10). This coding was used to gain an understanding on the health care utilization and the mortality rate for individuals diagnosed with FAS, as well as to estimate the associated health care costs in Canada for the most recent available fiscal year (2008–2009).

**Methods:**

Health care utilization data associated with a diagnosis of FAS were directly obtained from the Canadian Institute for Health Information (CIHI). Mortality data associated with a diagnosis of FAS were obtained from Statistics Canada.

**Results:**

The total direct health care cost of acute care, psychiatric care, day surgery, and emergency department services associated with FAS in Canada in 2008–2009, based on the official CIHI data, was about $6.7 million. The vast majority of the most responsible diagnoses, which account for the majority of a patient’s length of stay in hospital, fall within the ICD-10 category *Mental and Behavioural Disorders (F00*–*F99)*. It was evident that the burden and cost of acute care hospitalizations due to FAS is increasing −1.6 times greater in 2008–2009, compared to 2002–2003. The mortality data due to FAS, obtained from Statistics Canada (2000–2008), may be underreported, and are likely invalid.

**Discussion:**

The official data on the utilization of health care services by individuals diagnosed with FAS are likely to be underreported and therefore, the reported cost figures are most likely underestimated. The quantification of the health care costs associated with FAS is crucial for policy developers and decision makers alike, of the impact of prenatal alcohol exposure, with the ultimate goal of initiating preventive interventions to address FASD.

## Introduction

Maternal alcohol consumption during pregnancy is an established cause of Fetal Alcohol Spectrum Disorder (FASD), a group of disorders where alcohol exposure causes congenital damage to the central nervous system and other systems and organs of the fetus that subsequently leads to a number of adverse health consequences. FASD is a non-diagnostic umbrella term that covers several alcohol-related medical diagnoses, including: Fetal Alcohol Syndrome (FAS), Partial Fetal Alcohol Syndrome (pFAS), Alcohol-Related Neurodevelopmental Disorder (ARND), and Alcohol-Related Birth Defects (ARBD).

FASD is associated with a vast number and wide range of health conditions [1]–[7] and an increased mortality rate, as compared to the general population [1], [3], [6], [7]. As a result of the increased morbidity, FASD is a substantial burden to society in relation to the health care costs involved in caring for affected individuals.

In a recent review conducted by the authors of this study [8], [9], it was found that there are only a few studies that have assessed the economic impact of FAS/FASD in Canada [10]–[12] and the US [13]–[24]. Health care-specific FAS/FASD cost estimates have been done for Sweden [25], South Africa [26] and the US [27] only. Brief estimates that have included health care as a cost driver have also been completed for the Atlantic provinces of Canada [28] and various states in the US [29]–[33], as well as the US as a whole [34].

In 1996–1997, for the State of North Dakota in the US, Klug and Burd [35] estimated that the prevention of one case of FAS each year for 10 years would result in outpatient and inpatient health care costs savings of $129 thousand US dollars (USD), and the prevention of one case each year for 20 years would decrease these expenditures by $492 thousand USD.

Despite the few existing Canadian FASD cost studies, performed on an individual level with a cross-sectional study design [10], [11], very little is known about the utilization of health care services by individuals with FAS and the associated costs in Canada. As the prevalence/incidence of FAS in Canada remains uncertain, establishing the burden of health care and economic cost for FAS is challenging.

The purpose of this study was to determine the utilization of health care by individuals diagnosed with FAS and to estimate the associated direct cost in Canada for the most recent available fiscal year (2008–2009) by using official medical data collected by Canadian Institute for Health Information (CIHI) across Canada.

Given that FAS is the only FASD-related diagnosis coded in the *International Classification of Diseases* (ICD): in the ICD, Version 9, clinical modification (ICD-9-CM), *Alcohol affecting foetus or newborn via placenta or breast milk* 760.71, and in the ICD, Version 10 (ICD-10), *Fetal alcohol syndrome (dysmorphic)* Q86.0, it is the only FASD-related diagnosis that can be extracted from health records/databases.

Mortality data were also sought in order to further the understanding of the mortality rate in FAS-affected individuals.

## Methods

### Ethics Statement

Not applicable.

### Sources of Health Care Utilization Data

Health care utilization data of individuals diagnosed with FAS were obtained from CIHI (http://www.cihi.ca). CIHI is a government funded non-profit agency that collects, consolidates and provides unbiased, credible and comparable Canadian health care information. CIHI manages a large number of Canadian health databases, and receives information/data from the Canadian government, as well as Canadian hospitals.

There can be up to 25 diagnoses recorded on a patient’s medical chart in Canada. The most responsible diagnosis (MRD) is defined as the single diagnosis that describes the most significant condition of the patient that is responsible for his or her stay in hospital. In a case where multiple diagnoses may be classified as the MRD, coders are instructed to code the diagnosis responsible for the greatest length of stay.

The most recent available data (from April 1^st^, 2008 to March 31^st^, 2009) on health care service utilization, where a diagnosis of FAS was captured as either the MRD, secondary or other diagnosis, were searched for in the following databases: the Discharge Abstract Database (DAD), the Hospital Morbidity Database (HMDB), the National Ambulatory Care Reporting System (NACRS), the Ontario Mental Health Reporting System (OMHRS) and the Hospital Mental Health Database (HMHDB). It was requested that the data be broken down by gender, by age group (0–14, 15–29, 30–44, 45–59, 60–69, 70–79, and 80+), and by each province/territory.

#### Acute inpatient care

The DAD contains the number of acute care hospitalizations and hospital days for all provinces/territories, whereas the HMDB contains adult inpatient data for Quebec only.

#### Psychiatric care

The DAD contains the number of psychiatric hospitalizations and hospital days for all provinces/territories, except for the province of Ontario. Adult inpatient mental health services, as either acute or psychiatric care, for the province of Ontario is available from OMHRS. The HMHDB contains data from five specialty psychiatric facilities (from Manitoba, Saskatchewan and Prince Edward Island) that do not report to the DAD.

#### Day surgery

The DAD contains the number of day surgery hospitalizations and number of hours for all provinces/territories, except for Ontario, Quebec, Alberta and a small part of Nova Scotia, while the NACRS contains day surgery data for Ontario and a small part of Nova Scotia. Thus, day surgery data represents all provinces/territories except for Alberta and Quebec.

#### Emergency department

The NACRS contains the number of emergency department visits and the number of hours for Ontario only.

It is important to note that each hospitalization does not represent a separate case of FAS, as one case can be counted multiple times (once per visit).

### Sources of Cost Data

The average cost per day at acute care and psychiatric hospitals and the average cost per hospitalization for day surgery and emergency department visits by province/territory, as well as for all of Canada were estimated by CIHI upon the request of the authors using data from the Canadian MIS Database (CMDB) for 2008–2009 (Table 1).

**Table 1:** Average cost per day/visit by expense type on a provincial/territorial and national level for Canada, 2008–2009.

	Average costper day inacute care	Average costper day inpsychiatric care	Average costper visit inday surgery	Average costper visit inemergencydepartment
	($)	($)	($)	($)
Alberta	759.65	463.48	219.38	127.36
British Columbia	740.54	424.68	110.22	166.17
Manitoba	564.67	349.57	206.87	166.24
New Brunswick	607.33	576.01	130.66	133.95
Newfoundland	675.51	445.39	164.59	98.25
Nova Scotia	657.59	379.60	107.97	126.70
Ontario	674.73	394.61	163.86	191.02
Prince Edward Island	569.53	363.13	NA	90.48
Quebec	NA	NA	NA	NA
Saskatchewan	725.73	347.18	101.72	168.29
Northwest Territory	836.92	691.46	NA	163.92
Nunavut	NA	NA	NA	NA
Yukon Territory	647.99	NA	NA	94.68
Canada	684.49	412.58	157.48	163.96

NA - not available.

Source: CIHI, 2011 (CMDB).

### Cost Calculations

The total cost of acute inpatient and psychiatric care in Canada for 2008–2009 was calculated by taking the average cost per day in Canada for acute inpatient and psychiatric care and multiplying the respective numbers by the number of hospital days associated with FAS in acute inpatient and psychiatric care, respectively.

In regard to the cost calculations for those FAS associated day surgery hospitalizations or emergency department visits, the average cost per visit for day surgery in Canada and the average cost per visit for emergency department in Ontario were multiplied by the number of hospitalizations in the respective categories.

It should be noted that CIHI suppresses cells with fewer than five cases for hospitalizations, hospital days, and/or visits in order to ensure the confidentiality of the data. In instances where there were fewer than five cases, a midpoint of 2.5 was imputed on those cells.

### Sources of Morality Data

FAS mortality data were available from 2000 to 2008 and obtained from Statistics Canada by age group and gender [36].

The health care utilization and cost data, presented in this paper, are available from CIHI upon request, at a cost to the requester. The health care utilization data were received at an aggregated level and were anonymous.

Anonymous mortality data were obtained from Statistics Canada, which are freely available from the following website: http://www5.statcan.gc.ca/cansim/a01?lang=eng.

Due to the anonymous and aggregated nature of the analyzed data, it was not necessary to consult an institutional review board in regard to the current study.

## Results

The data by province/territory, as well as by gender was not possible to obtain from CIHI due to their privacy and confidentiality policies (cells with less than 5 cannot be reported in order to avoid residual disclosure of the identity of any of the parties involved). In addition, the search revealed that there were no cases with a diagnosis of FAS reported to the HMHDB.

### Health Care Utilization Data

In regard to the number of recorded hospitalizations and number of hospital days/hours for acute care, psychiatric care, and emergency department visits, the highest utilization rate among individuals diagnosed with FAS was observed among those 15 to 29 years of age, the second highest rate was among those from birth to 14 years of age (Table 2).

**Table 2:** Number of recorded hospitalizations and visits and hospital days, associated with a diagnosis of FAS and the associated costs in Canada for 2008–2009.

Level of Care (Databaseof CIHI)	Age Groups (years)							Total Number of hospitali-zations/days	Costs ($)
	0–14	15–29	30–44	45–59	60–69	70–79	80+		
**Number of acute** **care hospitalizations** **(DAD, HMDB & OMHRS)**	256	249	103	21	6	<5	0	738	N/A
**Number of acute care** **hospital days** **(DAD, HMDB & OMHRS)**	2,911	3,628	973	356	32	74	0	7,974	5,458,123.26
**Number of psychiatric** **care hospitalizations** **(DAD & OMHRS)**	14	43	9	<5	0	0	0	69	N/A
**Number of psychiatric** **care hospital days** **(DAD & OMHRS)**	308	1,353	1,230	14	0	0	0	2,905	1,198,544.90
**Number of day** **surgery hospitalizations** * **(DAD & NACRS)**	26	9	5	5	0	0	0	42.5	6,692.90
**Number of emergency** **departments visits** **(ON only; NACRS)**	18	24	<5	0	0	0	0	44.5	8,500.39
**TOTAL**									6,671,861.45

* Data from Quebec and Alberta are not included.

N/A – not applicable.

Note. In instances where there were fewer than five cases (<5), a midpoint of 2.5 was imputed on those cells. As a result, there may be rounding errors after collapsing the numbers.

Source: CIHI, 2011 (DAD, HMDB, NACRS, OMHRS).

The rate of utilization dropped substantially in the age group 45 to 59, and after age 60 plus there were zero recorded cases for all levels of care, except for acute care hospitalizations.

The pattern is somewhat different for day surgery, where the highest utilization rate was in the age group from birth to 14 years of age, followed by the age group 15 to 29.

Acute inpatient care had the highest rate of utilization across all age groups, compared to psychiatric care even though the MRDs were overrepresented by mental and behavioural disorders (as will be discussed below).

Table 3 presents the percentage of records of the top MRDs among individuals diagnosed with FAS who used acute care, psychiatric care, day surgery, and emergency department services in Canada in 2008–2009. The list represents only 55% of all records with a diagnosis of FAS; the remaining 45% had MRDs where the percentage of records was negligible (i.e., <0.5%).

**Table 3:** Percentage of records of the top most responsible diagnoses (MRDs) among individuals diagnosed with FAS who used acute care, psychiatric care, day surgery, and emergency department services in Canada in 2008–2009.

ICD-10 Code	Most Responsible Diagnosis	Percentage of Records*
Q86.0	Fetal alcohol syndrome (dysmorphic)	7.1
F43.2	Adjustment disorders	6.9
F90.0	Disturbance of activity and attention	5.4
F29	Unspecified nonorganic psychosis	3.3
F20.9	Schizophrenia, unspecified	2.6
F91.9	Conduct disorder, unspecified	2.5
F32.9	Depressive episode, unspecified	2.1
P07.1	Other low birth weight	1.7
F32.2	Severe depressive episode without psychotic symptoms	1.6
F31.9	Bipolar affective disorder, unspecified	1.4
F39	Unspecified mood [affective] disorder	1.4
F91.3	Oppositional defiant disorder	1.3
K02.9	Dental caries, unspecified	1.3
F43.1	Post-traumatic stress disorder	1.2
F25.9	Schizoaffective disorder, unspecified	1.0
J18.9	Pneumonia, unspecified	1.0
R56.8	Other and unspecified convulsions	0.9
T39.1	Poisoning by 4-Aminophenol derivatives	0.9
F43.0	Acute stress reaction	0.8
F60.3	Emotionally unstable personality disorder	0.8
F19.1	Mental and behavioural disorders due to multiple drug use and use of psychoactive substances, harmful use	0.7
F23.9	Acute and transient psychotic disorder, unspecified	0.7
F25.2	Schizoaffective disorder, mixed type	0.7
F69	Unspecified disorder of adult personality and behaviour	0.7
F79.9	Unspecified mental retardation without mention of impairment of behaviour	0.7
F90.1	Hyperkinetic conduct disorder	0.7
R45.8	Other symptoms and signs involving emotional state	0.7
B24	Human immunodeficiency virus [HIV] disease	0.6
E10.1	Type 1 diabetes mellitus with ketoacidosis	0.6
F10.0	Mental and behavioural disorders due to use of alcohol, acute intoxication	0.6
F10.2	Mental and behavioural disorders due to use of alcohol, dependence syndrome	0.6
F32.3	Severe depressive episode with psychotic symptoms	0.6
F41.9	Anxiety disorder, unspecified	0.6
F94.1	Reactive attachment disorder of childhood	0.6
G40.9	Epilepsy, unspecified	0.6

* The MRDs listed above represents 55% of all the records with a diagnosis of FAS; Source: CIHI, 2011 (DAD, HMDB, NACRS).

Twenty-five out of the 34 MRDs listed (i.e., 74%) are coded within the major disease category *Mental and Behavioural Disorders (F00*–*F99)*. As can be seen in table 3, the leading MRD was *Adjustment Disorders*, meaning that more than 6.9% of individuals (approximately 1 in 15) diagnosed with FAS were admitted to either acute care, psychiatric care, for day surgery or to the emergency department primarily due to this condition in 2008–2009.

Only 7% of individuals accounted for by the DAD, the HMDB and the NACRS had an MRD of FAS.

Table 4 presents the percentage of records of the top most responsible DSM-IV diagnoses among individuals diagnosed with FAS who used adult inpatient mental health services (acute and psychiatric care) in Ontario in 2008–2009.

**Table 4:** Percentage of records of the top most responsible DSM-IV diagnoses among individuals diagnosed with FAS who used adult inpatient mental health services (acute and psychiatric care) in Ontario in 2008–2009.

DSM-IV Diagnosis	Most Responsible Diagnosis	Percentage of Records*
**Q1f**	Mood disorders	38.5
**Q1o**	Adjustment disorders	15.4
**Q1a**	Disorders of childhood/adolescence	7.7
**Q1b**	Delirium, dementia, and amnestic and other cognitive disorders	7.7
**Q1c**	Mental disorders due to general medical conditions	7.7
**Q1d**	Substance-related disorders	7.7
**Q1e**	Schizophrenia and other psychotic disorders	7.7
**Q1n**	Impulse-control disorders not elsewhere classified	7.7

* The MRDs listed above represents 100% of all the records with a diagnosis of FAS.

Source: CIHI, 2011 (OMHRS).

The listed MRDs with the corresponding DSM-IV codes represent 100% of all the records associated with a diagnosis of FAS. The leading MRD was *Mood Disorders*, meaning that more than 38% of adult inpatients diagnosed with FAS occupied designated mental health beds in Ontario in 2008–2009 and required inpatient mental health care due to this condition. It is necessary to draw attention to the fact that the DSM-IV MRDs discussed here represent adult inpatient mental health services in Ontario only, and are as such not completely generalizable to Canada as a whole.

It was estimated that the cost for acute care hospital days, psychiatric care hospital days, and day surgery hospitalizations associated with a diagnosis of FAS in Canada in 2008–2009 was approximately $5.5 million, $1.2 million, and $6.7 thousand, respectively. The cost of emergency department visits associated with a diagnosis of FAS in Ontario was $8.5 thousand in 2008–2009.

The total direct health care cost associated with FAS in Canada in 2008–2009, based on the official CIHI data, was about $6.7 million (Table 2).

The data from the current analysis were compared to data from a previous study completed by the current authors [37], which used the same methodology – the number of hospitalizations and hospital days in acute care hospitals were obtained from CIHI and the associated cost due to morbidity attributable to FAS in Canada in 2002–2003 was estimated. When comparing the results of the two studies, it can be seen that the number of acute care hospitalizations due to FAS decreased from 838 in 2002–2003 to 738 in 2008–2009. However, the number of hospital days increased from 4,484 in 2002–2003 to 7,974 in 2008–2009, meaning that every day 21 or 22 hospital beds were occupied by patients with FAS in Canada in 2008–2009. As a result of the increase in the number of hospital days, the cost for acute care hospital days also increased from over $3.5 million (adjusted for inflation for 2008 using the Bank of Canada inflation calculator (http://www.bankofcanada.ca/en/rates/inflation_calc.html) in 2002–2003 to almost $5.5 million in 2008–2009.

The 2002–2003 data (not shown) revealed that between the ages of 0 (birth) −14 and 15–29, males had a higher number of days spent in an acute care hospital (2,358), as compared to females (1,785) of the same age. However, between the ages 30–44 and 45–59 females had a higher number of days spent in an acute care hospital (202), as compared to males (130) of the same age.

### Mortality

Surprisingly, there was only one death due to FAS in 2001 (under the age of 1, male), one in 2002 (between 20–24 years of age, female), two in 2006 (both between 15–19 years of age, females), one in 2007 (between 40–44 years of age, female), and one in 2008 (between 25–29 years of age, female). In total, there were only six deaths (one male and five females; documented as being attributable to FAS) during the 9-year period (2000–2008), as reported by Statistics Canada [36].

## Discussion

Of the four categorical diagnostic entities of FASD, FAS was the only diagnosis in which official data on health care utilization could be obtained. Therefore, the burden and cost figures in the current study are limited to only the available diagnosed FAS cases that were listed in the diagnostic formulations for episodes of health care. In this regard, the likelihood that the burden and cost figures in this study are underestimates of the true cost impact of FAS on the health care system in Canada is probable and would increase if all FASD-related diagnoses were included. It is thought that FAS represents only 10–20% of cases of FASD [38], [39], with ARND being the largest category of affected individuals by about three to four cases of ARND for every one case of FAS [40]. To further attest to this point, the authors of the current study recently compiled a list of more than 300 disease conditions associated with FASD (Popova et al., *unpublished*), which is not reflected in the data obtained.

Furthermore, given the fact that FAS is not widely recognized by health care practitioners, it is quite likely that for some of these cases, where the individual has been officially diagnosed previous to their contact with health care services in 2008–2009, FAS was not recorded in the medical chart if the precipitating event leading to care was not attributed to FAS. This again would result in an underestimate of the utilization and, in turn, the cost of health care, associated with FAS.

In order to estimate the error rate in capturing cost of care data for FAS due to inadequate diagnoses, we used the following assumptions: 1) the crude prevalence of FAS in Canada is 1 per 1,000 (0.1% [38]), and 2) the total population of Canada in 2009 was 33.7 million [41]. Therefore, there were approximately 33,730 people with FAS in Canada in 2009.

As reported in this study, there were 783 acute care hospitalizations and emergency department visits in 2008–2009. Therefore, the rate of hospitalization among the 33,730 people with FAS in Canada was about 2.3% during this time period.

According to official data obtained from CIHI, the rate of acute care hospitalizations and emergency room visits among the general population of Canada was about 8.3% (2.8 million hospitalizations in acute care facilities) in 2008–2009 [42]. Therefore, the rate of acute care hospitalizations among individuals with FAS was 3.6 times lower than that of the general population of Canada. This is unrealistic, given that the rate of morbidity among individuals with FAS is much higher, compared to that of the general population, as mentioned above.

Using the most conservative assumption that the rate of utilization of people with FAS is the same as in the general population, the cost of acute care, psychiatric care, day surgery, and emergency department services would be 3.6 times greater - about $24.0 million per year. Using the assumption that the rate of utilization among FAS individuals is as twice as high as that of the general population, the estimated cost of health care would double −$48.0 million per year in Canada.

The FAS specific mortality data, obtained from Statistics Canada [36], reported only six deaths attributable to FAS during the 9-year period (2000–2008). Some studies have reported that individuals with FASD have increased mortality rates as compared to the general population [1], [3], [6], [7], which is not demonstrated in the official data obtained from Statistics Canada. It is likely that FASD mortality rates are underreported since mortality in newborns, infants and young children most commonly occur before a diagnosis of an FASD can be made, as well as the fact that underdiagnosis ultimately leads to the underreporting. Thus, the rates of attributable mortality are likely invalid and should be the focus of an effort to improve case identification by provincial/territorial Medical Examiners across Canada.

While it is not possible to accurately estimate the error rate in capturing cost of care data for FAS due to inadequate diagnoses, it is realistic to suggest that these costs represent a modest fraction of the actual cost of health care for FAS. Despite this limitation, the results of this study have confirmed that FAS is a significant burden to the already over-burdened health care system in Canada. Moreover, the results revealed that the burden and cost of acute care hospitalizations due to FAS is increasing −1.6 times greater in 2008–2009, as compared to 2002–2003 (adjusted for inflation).

An important finding of the current study is that the vast majority of the MRDs among individuals with FAS fall within the ICD-10 category *Mental and Behavioural Disorders (F00*–*F99).* This finding highlights that there is a potential demand for psychiatric and psychological health care services in this population. It also stresses the need for prevention and early interventions to be implemented, in order to minimize the manifestation of such disorders in FASD-affected individuals.

This study draws attention to three main concerns in regard to FASD. First, there is an urgent need for increased capacity to provide prenatally alcohol exposed individuals with a proper diagnosis; second, doctors must be trained to both recognize, and diagnose the full range of FASD diagnoses; and third, a better recording system that will allow for the full range of FASD diagnoses to be recorded (i.e., an incorporation of the full range of FASD diagnoses to be coded in the ICD).

By improving the capture rate of FASD and by providing early and accurate diagnoses, efforts to prevent the secondary disabilities and decrease the health care utilization rate of individuals affected by prenatal alcohol exposure would be facilitated. In addition, early diagnosis has the potential to increase the use of substance abuse treatment for mothers and to prevent recurrent cases of FASD.

FASD is largely preventable. An understanding of the health care cost that is associated with this group of preventable disorders will hopefully enlighten policy and decision makers alike of the burden that FASD is on the Canadian health care system, as well as all of society. It is hoped that by informing these key players of the economic impact that FASD has on society, that funding and support for prevention initiatives will soon follow, with the ultimate result being a reduction in the economic burden of FASD.

It is important to point out that the health care cost associated with FASD is only one facet of a full and comprehensive cost estimate. Other components to consider, for example, but not limited to, are law enforcement, special education, social services, and indirect costs (productivity losses of FASD-affected individuals and their caregivers).

In conclusion, these cost figures, as a powerful argument, should not be misused for the further stigmatization of mothers with alcohol dependence, but rather, they should be used as a strong scientific evidence base demonstrating the cost of health care and utilization requirements for policy makers formulating policies on FASD.
